# Solution-processed near-infrared Cu(In,Ga)(S,Se)_2_ photodetectors with enhanced chalcopyrite crystallization and bandgap grading structure via potassium incorporation

**DOI:** 10.1038/s41598-021-87359-9

**Published:** 2021-04-09

**Authors:** Joo-Hyun Kim, Hyemi Han, Min Kyu Kim, Jongtae Ahn, Do Kyung Hwang, Tae Joo Shin, Byoung Koun Min, Jung Ah Lim

**Affiliations:** 1 National Agenda Research Division, Korea Institute of Science and Technology, Seoul, 02792 Republic of Korea; 2 Center for Opto-Electronic Materials and Devices, Korea Institute of Science and Technology, Seoul, 02792 Republic of Korea; 3 Department of Nano and Information Technology, KIST School, Korea University of Science and Technology (KUST), Daejeon, 34113 Republic of Korea; 4 UNIST Central Research Facilities, Ulsan National Institute of Science and Technology, Ulsan, 44919 Republic of Korea; 5 Graduate School of Energy and Environment, Korea University, Seoul, 02841 Republic of Korea

**Keywords:** Optoelectronic devices and components, Electronic devices

## Abstract

Although solution-processed Cu(In,Ga)(S,Se)_2_ (CIGS) absorber layers can potentially enable the low-cost and large-area production of highly stable electronic devices, they have rarely been applied in photodetector applications. In this work, we present a near-infrared photodetector functioning at 980 nm based on solution-processed CIGS with a potassium-induced bandgap grading structure and chalcopyrite grain growth. The incorporation of potassium in the CIGS film promotes Se uptake in the bulk of the film during the chalcogenization process, resulting in a bandgap grading structure with a wide space charge region that allows improved light absorption in the near-infrared region and charge carrier separation. Also, increasing the Se penetration in the potassium-incorporated CIGS film leads to the enhancement of chalcopyrite crystalline grain growth, increasing charge carrier mobility. Under the reverse bias condition, associated with hole tunneling from the ZnO interlayer, the increasing carrier mobility of potassium-incorporated CIGS photodetector improved photosensitivity and particularly external quantum efficiency more than 100% at low light intensity. The responsivity and detectivity of the potassium-incorporated CIGS photodetector reach 1.87 A W^−1^ and 6.45 $\times$ 10^10^ Jones, respectively, and the − 3 dB bandwidth of the device extends to 10.5 kHz under 980 nm near-infrared light.

Photodetectors, which convert optical into electrical signals, play a key role in current information technology^1–3^. Near-infrared (NIR) photodetectors, which sense photons with wavelengths longer than 750 nm, have gained much attention because these photons have long propagation distances and low attenuation in bio-tissues^1–3^. To date, crystalline silicon (Si) has dominated various optoelectronic devices owing to its superior performance and stability^4^. Traditional Si-based materials respond to broadband light ranging from 300 to 1100 nm, but they need to be applied to photoactive layers with thick junctions (*t* > 200 μm)^5^. Recently, Cu(In,Ga)(S,Se)_2_ (CIGS), perovskite, and organic semiconductors have been identified as potential substitutes for thin film NIR photoactive layers^1–3,6–8^. Among these photoactive materials, CIGS is one of the most promising materials for commercialization because of its high photon-to-electron conversion efficiency as well as environmental stability. In particular, CIGS films possess variable bandgap profiles ranging from 1.0 to 1.7 eV across their thickness depending on their penternary composition distributions^9–12^. This is advantageous in NIR photodetector applications.

In previous studies, CIGS photodetectors operating in a wide wavelength range from visible to NIR have been successfully demonstrated by Qiao et al.^6^. They proved that photodetectors based on CIGS heterojunctions have excellent NIR photoresponse performance with a responsivity (*R*) of up to 1.18 A W^−1^ at 808 nm, which also exerted a piezophototronic effect. However, the CIGS-based heterojunction films were fabricated using the co-evaporation method in vacuum-based equipment, which incurred a high production cost. Recently, considerable progress has been made in the development of multiple precursor coatings and post-chalcogenization processes based on benign solvents, providing manufacturability^12–14^. In addition, the power conversion efficiency of solution-processed CIGS photovoltaic devices has reached over 15.0%^13–18^. However, solution-processed CIGS NIR photodetectors have not been extensively investigated.

CIGS films often possess double bandgap grading structures that have the minimum bandgap (*E*_g,min_) near the surface and an bandgap (*E*_g_) that increases toward the bottom surface^19–21^. The *E*_g,min_ region contributes to the maximization of the absorption wavelength range while the bandgap grading toward the bottom CIGS surface contributes to the increase of the charge carrier collection and reduction of the carrier recombination attributed to the quasi-electric field^20,21^. The solution process employs precursor solutions where the relevant metal elements are all mixed together^9^. This makes it difficult to achieve the desired composition distributions and hence bandgap grading structure. In addition, during post-chalcogenization, Se is supplied from the top surface, which limits Se penetration and hence results in limited chalcopyrite grain growth. It is presumed that a wide *E*_g,min_ region and large grain formation are necessary to obtain highly performing NIR photodetectors. Here, we present a promising approach for fabricating CIGS NIR photodetectors based on a solution-processed CIGS photoactive layer. To further extend the light absorption range to the NIR region, a unique potassium (K)-incorporation in the bulk CIGS films is introduced to form a bandgap grading structure with a wide *E*_g,min_ region by promoting Se penetration. This wide *E*_g,min_ region results in a wide width of space charge region (*W*_SCR_) where the charge collection efficiency approaches 1. This process also facilitates chalcopyrite crystal and grain growth, leading to improved carrier mobility. We demonstrate that the fabricated K-incorporated CIGS photodetectors based on this process exhibit improved photoresponse characteristics with a high sensitivity of *R* = 1.87 A W^−1^, detectivity of D* = 6.45 $\times$ 10^10^ Jones, and fast response rate of 0.02 s, which are obtained at far NIR 980 nm and comparable to the performances from other CIGS photodetectors^6,22^. The external quantum efficiency (EQE) of the K-incorporated CIGS device exceeds 100% under very low NIR light power, which might be due to the photoconductive gain effect induced by the hole carrier tunneling from ZnO under reverse bias condition combined with high hole mobility. Consequently, we suggest that the K-incorporated CIGS films have great potential for NIR photodetection applications.

## Results and discussion

**Figure 1:**
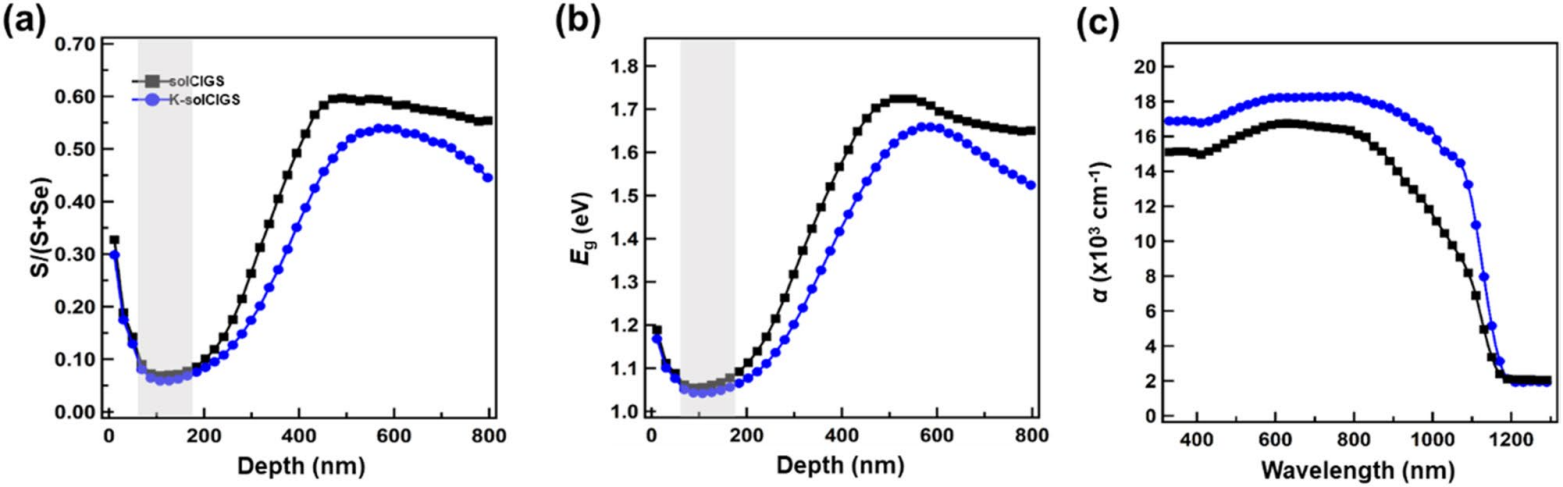
(**a**) Relative elemental ratio of S/(S + Se) and (**b**) bandgap energy of solCIGS and K-solCIGS thin films; modified with permission from ref 14, Copyright 2020, WILEY–VCH. The gray colored areas show the minimum bandgap (*E*_g,min_) region. (**c**) Optical absorption coefficient (*α*) spectra of solCIGS and K-solCIGS thin films.

The K-incorporation process in the CIGS photodetector fabrication is schematically shown in Supplementary Fig. S1^14^. The Cu-In-Ga (CIG) precursor and potassium fluoride (KF) solution were formed by dissolving copper nitrate hydrate (Cu(NO_3_)_2_·xH_2_O), indium nitrate hydrate (In(NO_3_)_3_·xH_2_O), and gallium nitrate hydrate (Ga(NO_3_)_3_·xH_2_O), and KF in methanol, respectively. A six-layered CIG oxide (CIGO_x_) film was fabricated by applying the precursor coating six times and the K-layer was applied on the third CIG oxide layer, followed by post-chalcogenization comprising selenization and sulfurization. The S and Se compositions were tuned by sulfurization and selenization, which were performed at elevated temperatures under S and Se vapor formed by the supply of H_2_S gas and sublimation of Se pellets, respectively. Hereafter, the solution-processed CIGS films with and without K-incorporation are referred to as the K-solCIGS and solCIGS films, respectively. To investigate the composition in the CIGS films, dynamic secondary-ion mass spectroscopy (D-SIMS) with Cs^+^ ion-assisted sputtering was performed. The atomic concentrations of Cu, Na, and K and In, Ga, S, and Se were obtained by atomic absorption spectroscopy (AAS) and inductively coupled plasma-optical emission spectroscopy (ICP-OES) measurements, respectively. The relative sensitivity factor (RSF) was calculated by integrating the D-SIMS profile and dividing this value by the AAS or ICP-OES value. The atomic concentration profile was obtained by dividing the D-SIMS profile by the RSF value, from which the S/(S + Se) and *E*_g_ profiles were estimated^23^, as shown in Fig. 1a,b, respectively. There was an increase in Se and decrease in S in the K-solCIGS film, leading to a decreased S/(S + Se) profile with a wide minimum region in the K-solCIGS film compared to the profile from the unmodified solCIGS film. Notably, the diffusion of K toward the bottom CIGS film during chalcogenization accelerated the Se uptake toward the bottom CIGS film owing to the K–Se chemical affinity. During chalcogenization, Se and S reacted preferentially with In and Ga, respectively, and In/Se and Ga/S often segregated to the top and bottom, respectively. In addition, S was enriched by the final sulfurization. These composition distributions led to a typical bandgap grading structure with the *E*_g,min_ region (gray region) near the top, referred to as the notch region, and an increased *E*_g_ toward the bottom CIGS film, referred to as the notch depth. The *E*_g_ profile of the K-solCIGS film has a wider notch region than the solCIGS film, which is attributed to the decrease in S/(S + Se). This result clearly shows that the incorporation of K in the K-solCIGS film resulted in the formation of a bandgap grading structure with a lower value compared to the conventional CIGS film. Figure 1c shows the absorption coefficient (α) of the solCIGS and K-solCIGS films. The CIGS films exhibited broad absorption spectra from 300 to 1200 nm with a large absorption coefficient (*α* ≈ 10^4^ cm^−1^). The K-solCIGS film displayed a lower energy *E*_g_ for the same penetration depth (Fig. 1b), resulting in a pronounced improvement in the NIR region absorption coefficient. This makes the K-solCIGS more suitable for NIR photoactive layers.

**Figure 2:**
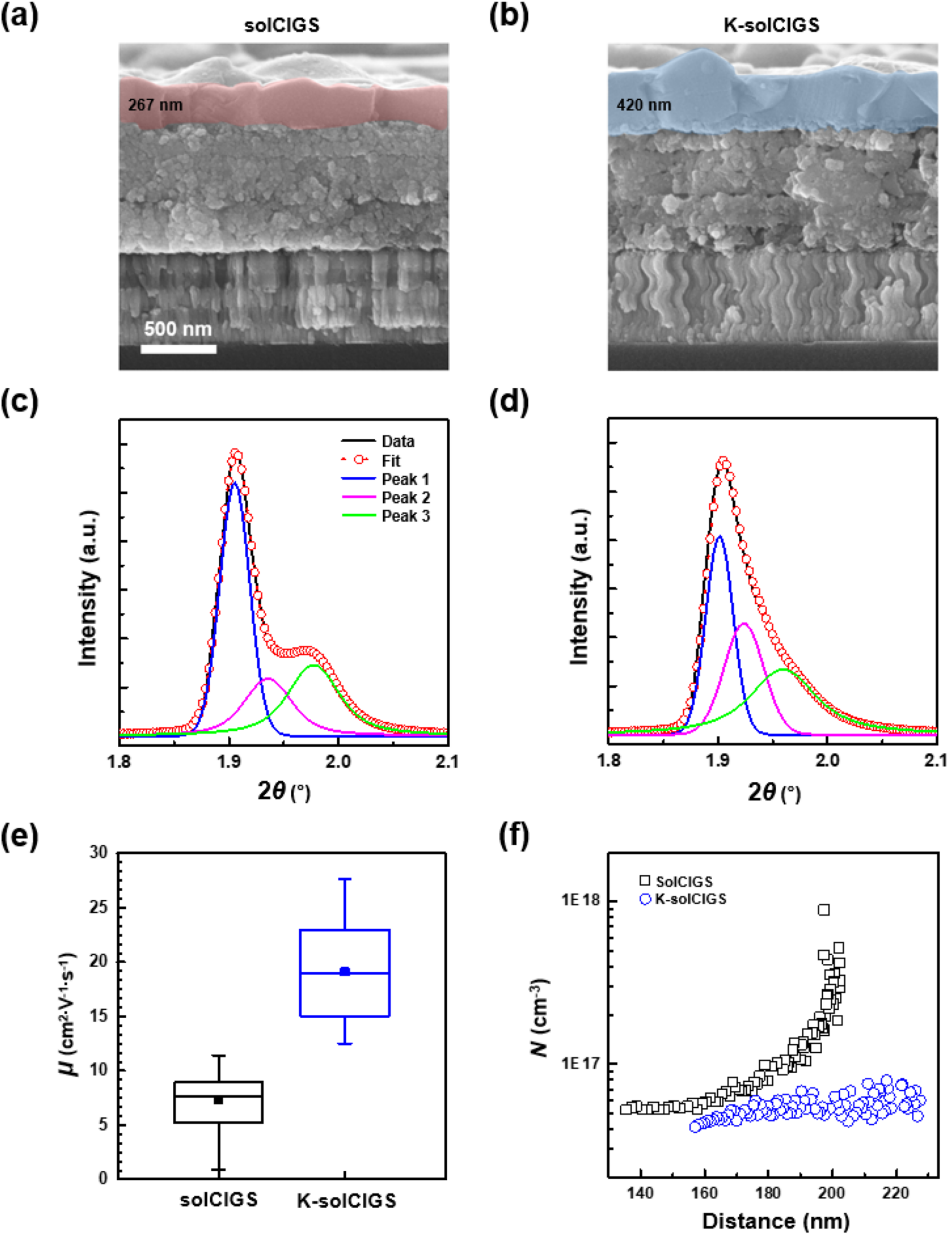
(**a**,**b**) Cross-sectional SEM images of (**a**) solCIGS and (**b**) K-solCIGS thin films. Colored areas denote upper layer regions. (**c**,**d**) Circular averaged 1D GI-WAXS profiles of (**c**) solCIGS and (**d**) K-solCIGS thin films. (**e**) Box plots, showing variation range of Hall mobility (*μ*) of solCIGS and K-solCIGS devices. (**f**) Spatial distributions of the charge carrier density, estimated by *C*‒*V* measurement of solCIGS and K-solCIGS devices; modified with permission from ref 14, Copyright 2020, WILEY–VCH.

The chalcopyrite morphology development was comparatively assessed by estimating the thickness of the upper layers of the solCIGS and K-solCIGS films as shown in the cross-sectional SEM images in Fig. 2a,b, respectively. The thicknesses of the solCIGS and K-solCIGS upper layers were 267 and 420 nm, respectively (Table 1). The thicknesses indicate that chalcopyrite grain growth in the K-solCIGS film was promoted in comparison to the solCIGS film. The elongated Se penetration seems to promote selenization and hence chalcopyrite grain growth. In addition to the grain growth, the crystal growth of the solCIGS and K-solCIGS films was evaluated using grazing-incidence wide-angle X-ray scattering (GI-WAXS) under various incidence angles of α_i_ = 0.14–0.7°. The representative 2D GI-WAXS patterns of the solCIGS and K-solCIGS films measured at α_i_ = 0.7° are shown in Supplementary Fig. S2, in which the applied incidence angle was much higher than the critical angle of CIGS film (α_c_ ≈ 0.13° at 18.986 keV) in order to obtain crystallographic information about the total thickness of the film. The characteristic diffraction peaks observed near 1.90, 3.10, and 3.65 Å^−1^, corresponding to the (112), (220), and (312) plane, respectively, were identified as the chalcopyrite CIGS phases^12,14,24^. The circular averaged 1D profiles obtained from 2D GI-WAXS patterns at α_i_ = 0.7°, especially enlarged around (112) diffraction peak, are shown in Fig. 2c,d. The (112) diffraction peak of K-solCIGS film can be deconvoluted with three peaks located at 1.900, 1.923, and 1.963 Å^−1^. These peak positions are slightly lower than those of solCIGS films (1.904, 1.936, and 1.978 Å^−1^, respectively), suggesting improved selenization in the K-solCIGS film^14^. Furthermore, the peak width (FWHM: full-width at half maximum) of the lowest angle peak (Peak 1) of K-solCIGS and solCIGS film is 0.0297 and 0.0324 Å^−1^, respectively, where the apparent crystal size estimated by Scherrer equation^25^ corresponds to 19.0 and 17.4 nm, respectively. The slightly larger crystals with Se rich composition reveal that K-solCIGS film is developed from facilitated selenization. Since CIGS grains comprise small crystals, indeed large crystals contribute to form large grains in K-solCIGS film. Figure 2e displays the range of Hall mobilities estimated for both the K-solCIGS and solCIGS films^26^. Typical solCIGS films show hole mobilities in the range of 0.9 to 11.4 cm^2^ V^−1^ s^−1^, while the K-solCIGS films have a 61.5% higher hole mobilities in the range of 12.5–27.6 cm^2^ V^−1^ s^−1^. Averaged hole mobility is 7.3 and 19.1 cm^2^ V^−1^ s^−1^ for solCIGS and K-solCIGS film, respectively (Table 1). This improved carrier mobility is attributed to the enhanced crystal and grain size in K-solCIGS by K-incorporation. The larger grains with reduced grain boundaries ensure the existence of percolation paths because the boundary and gaps suppress carrier transport and act as recombination centers^14^. In addition, the wide notch region and mitigated notch depth in the K-solCIGS bandgap grading structure also contributes to a decrease in the electron accumulation in the *E*_g,min_ region, and thus leads to a decreasing charge carrier recombination and increasing charge transport and collection^20,21^. Note that *W*_SCR_ is inversely related to the hole carrier density (*N*), estimated with capacitance‒voltage (*C*‒*V*) measurement as shown in the following equation^14^:

1 $${\text{W}}_{{{\text{SCR}}}} { = }\sqrt {\frac{{{2}\varepsilon_{{0}} \varepsilon_{{\text{r}}} {\text{(V}}_{{{\text{bi}}}} - {\text{V)}}}}{{{\text{q}}N}} } { ,}$$

where *q* is the elementary charge, *ε*_0_ and *ε*_r_ are the permittivity of vacuum and the dielectric constant of the device, respectively, and *V*_bi_ is the built-in potential. The larger *W*_SCR_ (158 nm) is obtained for K-solCIGS device than the *W*_SCR_ of 150 nm for solCIGS device. On the contrary, hole carrier density in K-solCIGS device is 4.11 × 10^16^, which is significantly reduced value compared to the 5.24 × 10^16^ from solCIGS device in over the measured distance ranges (Fig. 2f and Table 1). Hole transport and collection is facilitated in K-solCIGS device ascribed by increasing hole mobility, resulting in decreasing *N* in space charge region and increasing *W*_SCR_ in K-solCIGS device.

**Table 1:** Summary of upper layer thickness, Hall mobility, and charge carrier density (*N*) estimated by SEM analysis, and Hall effect and *C*‒*V* measurements, respectively.

	Upper layer thickness (nm)	Hall mobility (cm^2^ V^−1^ s^−1^)	*N* (cm^−3^)
solCIGS	267 ± 25	7.3 ± 2.5	5.24 × 10^16^
K-solCIGS	420 ± 38	19.1 ± 4.9	4.11 × 10^16^

**Figure 3:**
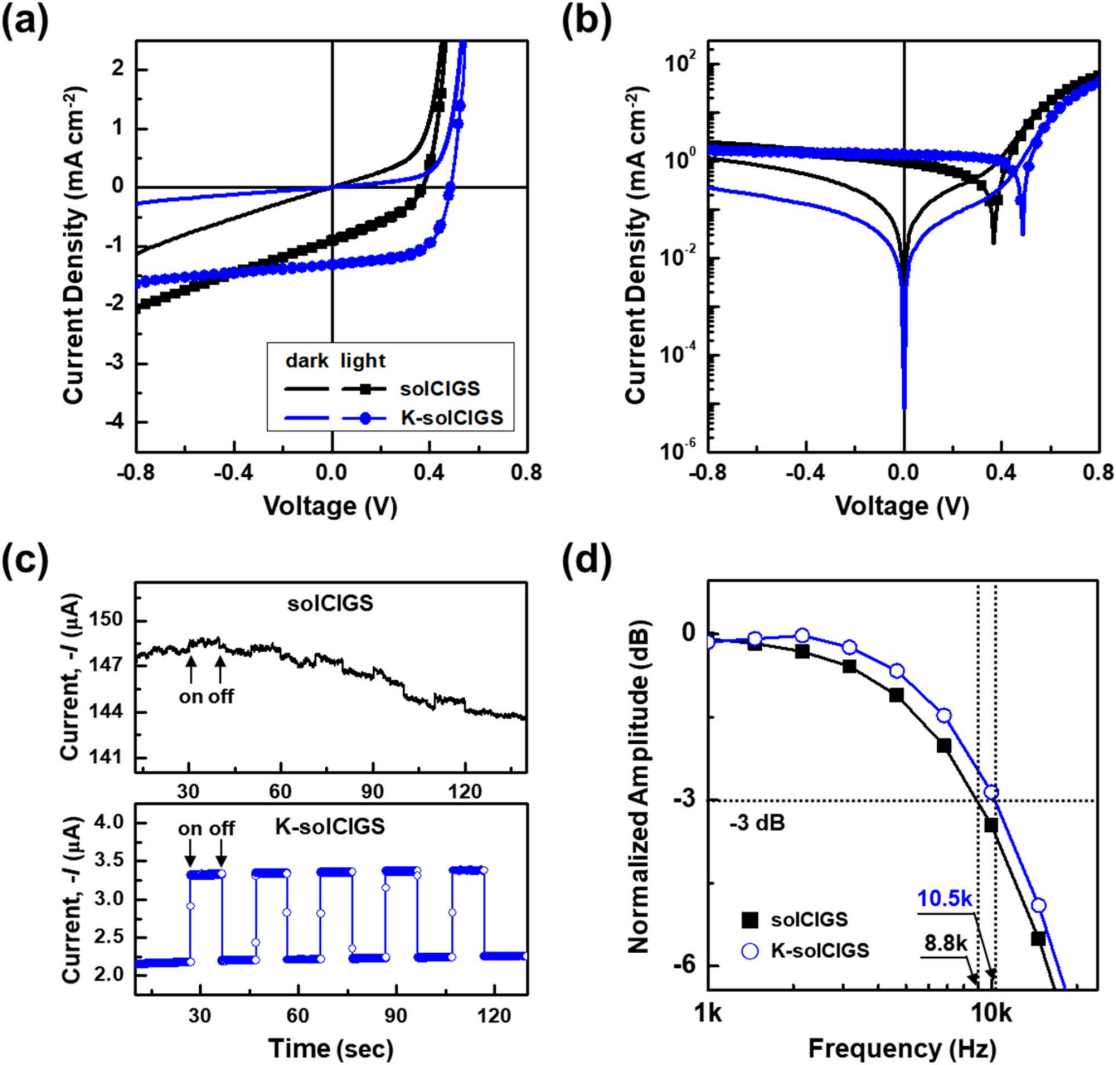
(**a**,**b**) Current density‒voltage (*J*‒*V*) characteristics of solCIGS and K-solCIGS photodiodes in the dark and under illumination at the NIR wavelength of 980 nm (*P*_IN_ = 31.4 mW cm^−2^). Note that the graphs in (**a**) and (**b**) show a linear plot and a semi-logarithmic plot of the *J*‒*V* characteristics, respectively. (**c**) Time-dependent photoresponse of solCIGS and K-solCIGS devices at − 0.4 V (*λ* = 980 nm, *P*_IN_ = 0.03 mW cm^−2^). (**d**) − 3 dB bandwidth comparison of solCIGS and K-solCIGS devices as a function of incident pulse laser frequency under NIR light of 980 nm.

The current density‒voltage (*J*‒*V*) characteristics of photodiodes based on solCIGS and K-solCIGS films measured between − 0.8 and + 0.8 V in the dark and under 980 nm illumination are shown in Figs. 3a,b. The current densities of the K-solCIGS device were greatly increased under illumination by 980 nm light compared to thoes of the solCIGS device, showing that the K-solCIGS film functions efficiently as a NIR photoactive layer in the device (Fig. 3a). In addition, the dark current (*I*_dark_) of the K-solCIGS device was distinctly lower than that of solCIGS device, resulting in a gentler slope of the K-solCIGS device under the reverse bias condition. This can be attributed to the better charge transport in the K-solCIGS film in accordance with the carrier mobility results (Fig. 2e). In addition, the K-solCIGS device exhibited enhanced photovoltaic behavior (Fig. 3b), implying that the K-solCIGS photodetector has further potential as a self-powered optoelectronic sensor without external energy consumption^22^. Besides, the *J‒V* characteristics of both photodiodes measured at 850 nm illumination were shown in Supplementary Fig. S3a,b, indicating that the K-solCIGS device has higher photocurrent and lower dark current than the solCIGS device in the NIR region regardless of wavelength.

Based on these results, the time-dependent photoresponse curves of the CIGS devices measured by the optical on/off modulation of the incident NIR light (*λ* = 980 nm, *P*_IN_ = 0.03 mW cm^−2^) at *V* = − 0.4 V (reverse bias condition) are depicted in Fig. 3c. Under repeated on/off switching of the light, the solCIGS device exhibited no photoresponse and a significant photocharging phenomenon, implying inefficient charge extraction due to local carrier accumulation. By contrast, very stable photocurrent signals with a rapid response and recovery time of 0.02 s were measured in the K-solCIGS device, even at a low light intensity of 0.03 mW cm^−2^. In addition to the current density and photoresponse properties, the photoelectrical bandwidth, which indicates the high-frequency response ability, was obtained by investigating the cut-off frequency of the photodiodes to the incident NIR pulse laser frequency. The K-solCIGS device exhibited an improved − 3 dB bandwidth of up to 10.5 kHz compared to the 8.8 kHz for the solCIGS device (Fig. 3d). These results confirm that the K-solCIGS photodiodes present an increased photocurrent and extended − 3 dB bandwidth as a result of the increased charge carrier mobility due to K-incorporation. Analogous improvement in photodetecting properties of K-solCIGS photodiode compared to the solCIGS photodiode is also confirmed at the 850 nm illumination condition (Supplementary Fig. S3c,d in the supplementary information).

**Figure 4:**
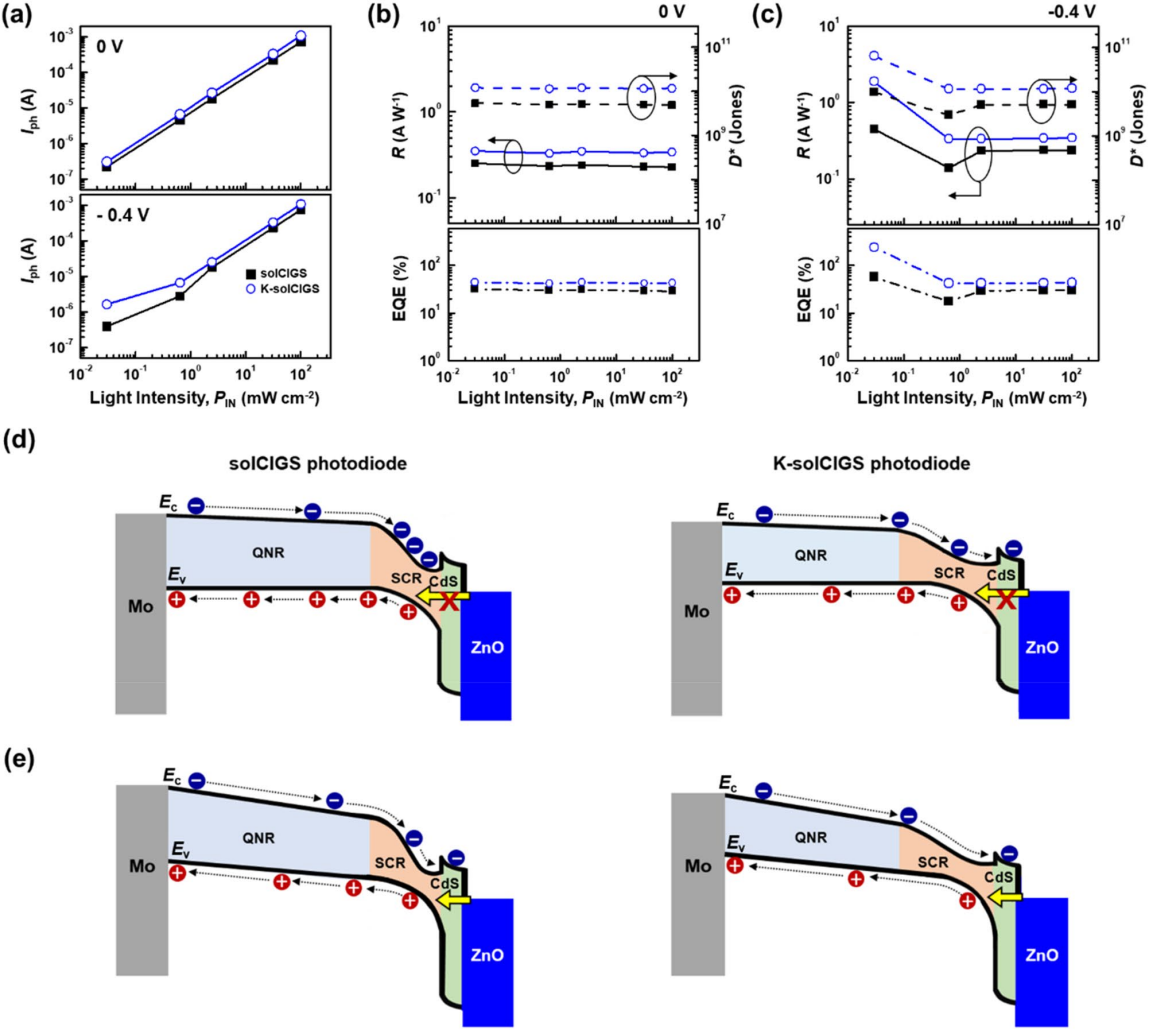
(**a**) Photocurrent (*I*_ph_) as a function of the light intensity (*P*_IN_) under NIR wavelength illumination of 980 nm at 0 V and − 0.4 V. (**b**,**c**) Responsivity (*R*), detectivity (D^*^), and EQE as functions of the light intensity (*P*_IN_) under NIR wavelength illumination of 980 nm at (**b**) 0 V and (**c**) − 0.4 V. (**d**,**e**) Schematic energy band diagrams of solCIGS and K-solCIGS photodiodes at low light intensity under (**d**) 0 V and (**e**) − 0.4 V (reverse bias condition). At 0 V, tunneling of hole does not occur. However, at − 0.4 V, holes are allowed to tunnel from ZnO to CIGS, as the yellow arrow indicates.

The light intensity-dependent photocurrents (*I*_ph_) of the solCIGS and K-solCIGS photodiodes in the absence of applied voltage (photovoltaic mode) and under the reverse bias condition of − 0.4 V (photoconductive mode) are plotted in Fig. 4a. Both devices exhibited an *I*_ph_ that increases linearly with the incident light intensity in photovoltaic mode operation, indicating the stable photoresponsivity of the devices. However, when the devices were operated under reverse bias conditions, the relationship deviated from linearity on the logarithmic scale below the light intensity of 10^−1^ mW cm^−2^, and the *I*_ph_ of the K-solCIGS photodiodes showed higher photocurrent values compared to the *I*_ph_ of the devices under photovoltaic mode operation at the same light intensity. This behavior might be due to the increased EQE of the device as a result of the photoconductive gain effect, which will be discussed later. The photoresponsivity (*R*), detectivity (D^*^), and EQE of the photodiodes were estimated from the *J–V* curves in Fig. 3 using the following equations^2,3^:

2 $$\it {\text{R}} = \frac{{{\text{I}}_{{{\text{light}}}} - {\text{I}}_{{{\text{dark}}}} }}{{P_{{{\text{IN}}}} }}{ = }\frac{{I_{{p{\text{h}}}} }}{{P_{{{\text{IN}}}} }}{, }$$

3 $$\it {\text{D}}^{*} = \frac{{{\text{RA}}^{{1/2}} }}{{{\text{S}}_{{\text{n}}} }} = \frac{{{\text{RA}}^{{1/2}} }}{{{\text{(2qI}}_{{{\text{dark}}}} {)}^{{1/2}} }}{, }$$

4 $$\it \it {\text{EQE}} = \frac{{{\text{(I}}_{{{\text{light}}}} - {\text{I}}_{{{\text{dark}}}} {\text{)/q}}}}{{P_{{{\text{IN}}}} {\text{/(h}}\nu {)}}}{ = }\frac{{{\text{I}}_{{{\text{ph}}}} {\text{/q}}}}{{P_{{{\text{IN}}}} {\text{/(h}}\nu {)}}}{, }$$

where *P*_IN_ is the incident optical power, *I*_light_, *I*_dark_, and *I*_ph_ denote the current under illumination, the dark current, and the photocurrent, respectively, *A* is the active area of the device, $S_{n} = \left( {2qI_{dark} } \right)^{1/2}$ is the noise current, *q* is the electron charge, *h* is the Planck constant, and $\nu$ is the incident light frequency. Figure 4b,c exhibit the characteristic properties of the devices at zero bias and − 0.4 V reverse bias condition. The photoresponse characteristics of the devices remained almost constant regardless of the incident light intensity at *V* = 0 V, while thoes of the K-solCIGS photodiodes showed much improved performance compared to the solCIGS photodiodes (Fig. 4b)^27^. Interestingly, as the light intensity decreased below 10^−1^ mW cm^−2^, the characteristic parameters of the devices under the reverse bias condition increased (Fig. 4c). This is consistent with the light intensity-dependent photocurrent result (Fig. 4a). In fact, the maximum *R*, D*, and EQE of the K-solCIGS photodiodes were 1.87 A W^−1^, 6.45 $\times$ 10^10^ Jones, and 237%, respectively under *P*_IN_ = 0.03 mW cm^−2^, while those of the solCIGS photodiodes were 0.45 A W^−1^, 9.65 $\times$ 10^9^ Jones, and 57%, respectively, under the same measurement conditions. This improvement in the K-solCIGS devices is primarily attributed to the combined effect of the reduced *I*_dark_ and increased *I*_light_ due to the significantly enhanced charge carrier transport and extraction properties of the K-solCIGS film. In addition, the formation of a wide *W*_SCR_ region could contribute to the performance improvement in the K-solCIGS photodiode. It can be reasonably inferred that a wider *W*_SCR_ region might be formed at the ZnO:Al interface of the K-solCIGS device compared to that of the solCIGS device, as shown in Fig. 4d,e. Because the charge collection efficiency in the *W*_SCR_ region approaches 1, the wide *W*_SCR_ formed in the K-solCIGS photodiode resulted in an improvement in the charge collection efficiency and higher photocurrent. Moreover, this trend of photoresponse characteristics was observed identically under irradiance wavelength of 850 nm (*P*_IN_ = 0.03 mW cm^−2^), giving a larger improvement with *R* from 0.11 up to 4.83 A W^−1^ and D* from 2.50 $\times$ 10^9^ to 1.67 $\times$ 10^11^ Jones under the reverse bias condition, as shown in Supplementary Fig. S3e,f in the supplementary information. These results are attributed to the higher spectral absorption capability of K-solCIGS films under 850 nm than 980 nm (Fig. 1c), resulting in enhanced photocurrent generation (Supplementary Fig. S3a,b). Therefore, this further indicates that the K-solCIGS NIR photodiode has a potential to be applied to practical near IR optoelectronic systems including quantum computing^28^, LiDAR (light detection and ranging) imaging systems^29^, and 3D ToF (time-of-flight) sensors, that mainly use NIR photons around 850 nm^30^.

It should be noted that the EQE of the K-solCIGS devices reached 237% under 980 nm illumination at the low light intensity of 0.03 mW cm^−2^, which is much higher than 100%. This indicates that the photocurrent can be amplified by the photoconductive gain effect at low light intensity under reverse bias condition^31–33^. We propose a possible mechanism for this amplifciation based on the band grading structure. Because the *W*_SCR_ region is formed near the ZnO:Al interface where light is exposed, the effect of charge separation is dependent on the light intensity. At low light intensity, the light absorption is limited near the top electrode surface, and most of the excitons are generated in the space charge region (Fig. 4d,e). In the photoconductive mode under reverse bias (*V* = − 0.4 V), in the *W*_SCR_ the photogenerated charge carriers are separated prior to recombination and charge carriers effectively drift to the electrodes under an external electric field (Fig. 4e). Also, large band bending leads holes to tunnel from ZnO to CIGS valence band, producing increasing photocurrent by the hole injection as described in Fig. 4e ^34^. Furthermore, the hole mobility in K-solCIGS film is much higher than the value in the solCIGS film (Fig. 2e). Therefore, injected holes are effectively transported to the electrode, producing EQE more than 100% in K-solCIGS devices at the low power light^35–39^. In contrast, under illumination at high power, the light absorption occurs in the quasi-neutral region beyond the *W*_SCR_ region. Most of the carriers photogenerated in the wide quasi-neutral region are recombined before they are collected to the electrodes, which leads to an EQE of less than 100%.

## Conclusion

In conclusion, K was incorporated into bulk CIGS precursor film. This approach enhanced the Se penetration, thereby improving the level of chalcogenization and tuning the bandgap grading structure simultaneously. The resulting efficient charge transport and reduced carrier recombination from the promotion of large grain formation and the wide bandgap notch region allowed the achievement of an increase in the charge carrier mobility to 27.6 cm^2^ V^−1^ s^−1^ and wide optical sensitivity of up to 1200 nm, respectively. Consequently, the photocurrent of the K-solCIGS photodetector was significantly increased and the dark current was reduced, giving rise to *R* and D* values of 1.87 A W^−1^ and 6.45 $\times$ 10^10^ Jones under 980 nm NIR light, respectively, which are far larger than the 0.45 A W^−1^ and 9.65 $\times$ 10^9^ Jones from the solCIGS photodetector. Moreover, the − 3 dB bandwidth of the K-solCIGS photodiode was extended to 10.5 kHz, compared to the 8.8 kHz of the solCIGS photodiode. This work offers new possibilities for CIGS optical devices as NIR photodetectors, possibly used as biosensor and information communications, in the future.

## Methods

### Preparation of CIGS films

Molybdenum (Mo) was DC-sputtered on soda-lime glass (SLG) substrate to fabricate a Mo layer with 500 nm thickness. The Cu-In-Ga precursor solution was formulated by dissolving Cu(NO_3_)_2_·xH_2_O (4.80 mM), In(NO_3_)_3_·xH_2_O (3.82 mM), and Ga(NO_3_)_3_·xH_2_O (1.93 mM) in methanol (8 ml) at 25 °C for 2 days. The poly(vinyl alcohol) (PVA) binder and KF solutions were prepared by stirring polyvinyl acetate (0.125 g mL^−1^) and KF in methanol (0.1 mol KF with respect to Cu 1 mol), respectively. Precursor and binder solutions were mixed and spin-cast on Mo-sputtered SLG at 2000 rpm for 40 s. The films were subsequently air-annealed in a box furnace at 300 °C for 30 min. Casting and annealing of the CIGO_x_ layer were repeated six times and CIGO_x_ film with 1 μm thickness was fabricated. The KF solution was dropped on the third CIGO_x_ layers. The CIGO_x_ film was chalcogenized, comprising sulfurization and selenization proceeded by supplying H_2_S gas and Se pellets, respectively in a tube furnace, to form Cu(In_(1−*X*)_Ga_*X*_)(S_*Y*_Se_(1−*Y*)_)_2_ (0 < x < 1 and 0 < y < 1).

### Composition and morphology characterization

Depth profiles of CIGS composition were obtained using D-SIMS (IMS 4FE7, Cameca). Cs^+^ ions were sputtered as primary ions (5.5 keV impact energy, 30 nA ion current, and 200 × 200 μm^2^ raster size) and positive ions were detected as secondary ions. Atomic concentrations of Cu, Na, and K were obtained by AAS (iCE3000, Thermo Fisher Sientific) and those of In, Ga, S, and Se were obtained by ICP-OES (iCAP6500 Duo, Thermo Fisher Sientific) measurement. The CIGS film was cut into a small specimen (5 mm × 5 mm) and then dissolved in a 3% HNO_3_ solution and was also dissolved in DI water, 2% HCI, and 1.6% aqua regia for the AAS and ICP-OES measurements, respectively. The optical absorption property of CIGS film was estimated from reflectance spectra (Cary 5000, Varian). The cross-sectional images of the CIGS thin films were obtained using SEM (Inspect F, FEI corp.). The hard X-ray 2D GIWAXS patterns were collected at the 6D beamline at the Pohang Accelerator Laboratory (PAL). Peak separation analysis was carried out using peak fit program with Pseudo-Voigt function. Hall mobility was measured by using a Hall effect measurement system (HMS-3000, ECOPIA).

### Fabrication and characterization of CIGS photodiodes

A CdS buffer layer was formed on CIGS by chemical bath deposition. CdSO_4_ (0.08 mM) was dissolved in DI water (440 mL) and NH_4_OH (1.63 mM) mixture, filling a bath. The CIGS thin film was dipped into the bath and reacted for 10 min to form CdS layer (50 nm). Intrinsic zinc oxide (i-ZnO, 50 nm) and aluminum-doped zinc oxide (Al:ZnO, 500 nm) were sequentially deposited by radiofrequency sputtering to form a window layer. Ni (50 nm) and Al (500 nm) were e-beam evaporated through stainless-steel masks to form a top electrode. The effective area of devices was determined to be 0.25 cm^2^. All of the static electrical and photoelectric measurements of our devices were carried out using a semiconductor parameter analyzer (4156C, Agilent) in the dark and under NIR LD (laser diode) illumination of 980 nm (L980P030, Thorlabs) and 850 nm (L850P030, Thorlabs). The optical intensities of incident NIR lights were modulated by a laser diode controller (LDC205C, Thorlabs) and verified using a standard Si photodiode (DET10A, Thorlabs). The photoelectric bandwidth was measured using a function generator (33120A, Keysight Technology), a low-noise-current preamplifier (SR570, Stanford Research Systems), and a digital signal processing (DSP) lock-in amplifier (SR830, Stanford Research Systems). To calculate the specific detectivity (D*), the noise power spectral densities were measured using a low-noise-current preamplifier (SR570) and a FFT servo spectrum analyzer (Advantest R9211B, Advantest co., ltd.).

## Supplementary Information

Supplementary Information.

## Data Availability

The data that supports the findings of this study are available within the article and supplementary material.
