# Supplementary material for: Solution-processed near-infrared Cu(In,Ga)(S,Se)2 photodetectors with enhanced chalcopyrite crystallization and bandgap grading structure via potassium incorporation
Source: Sci Rep. 2021 Apr 9;11:7820. doi: 10.1038/s41598-021-87359-9 (PMC8035197; doi:10.1038/s41598-021-87359-9)
Supplement: Supplementary file 1 — Supplementary Information. [file 41598_2021_87359_MOESM1_ESM.docx]

*Supplementary Material*

**Solution-Processed Near-Infrared Cu(In,Ga)(S,Se)_2_ Photodetectors with Enhanced Chalcopyrite Crystallization and Bandgap Grading Structure via Potassium Incorporation**

*Joo-Hyun Kim,^1^ Hyemi Han,^2^ Min Kyu Kim,^1^ Jongtae Ahn,^2^ Do Kyung Hwang,^2,3^ Tae Joo Shin,^4^ Byoung Koun Min,^1,5,a)^ and Jung Ah Lim^2,3,a)^*

^1^National Agenda Research Division, Korea Institute of Science and Technology, Seoul 02792, Republic of Korea

^2^Center for Opto-Electronic Materials and Devices, Korea Institute of Science and Technology, Seoul 02792, Republic of Korea

^3^Department of Nano and Information Technology, KIST School, Korea University of Science and Technology (KUST), Daejeon 34113, Republic of Korea

^4^ UNIST Central Research Facilities, Ulsan National Institute of Science and Technology, Ulsan 44919, Republic of Korea

^5^Graduate School of Energy and Environment, Korea University, Seoul 02841, Republic of Korea

a) E-mail: [bkmin@kist.re.kr](mailto:bkmin@kist.re.kr); and [jalim@kist.re.kr](mailto:jalim@kist.re.kr)


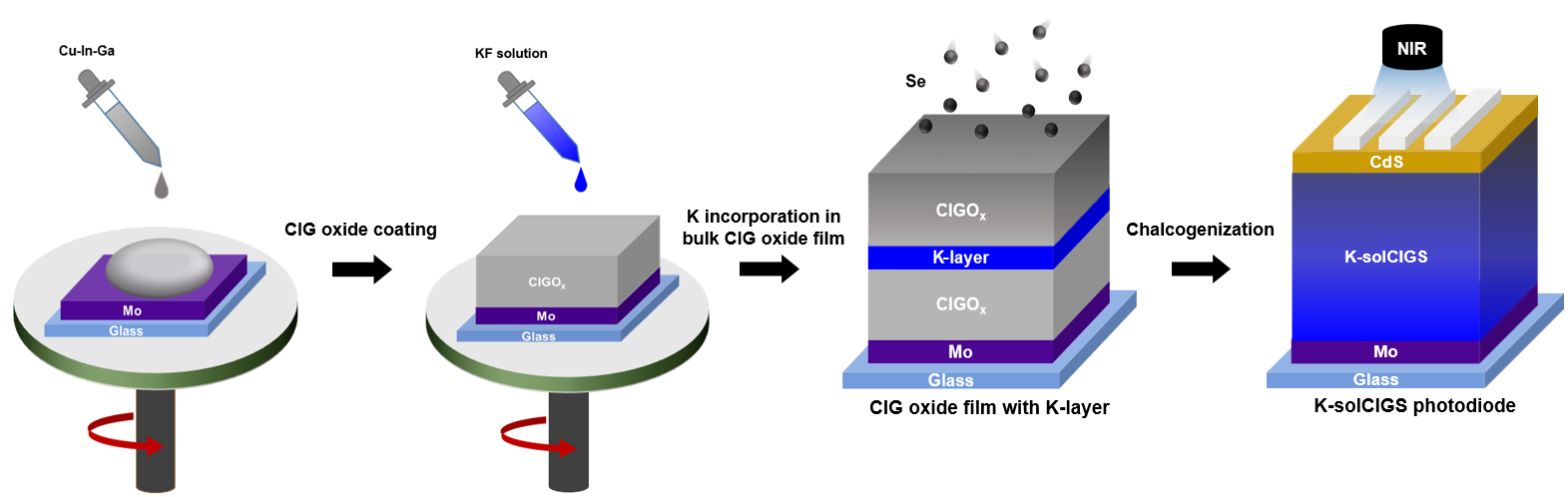


**Figure S1.** Schematic depiction of the K-solCIGS photodiode fabrication process.


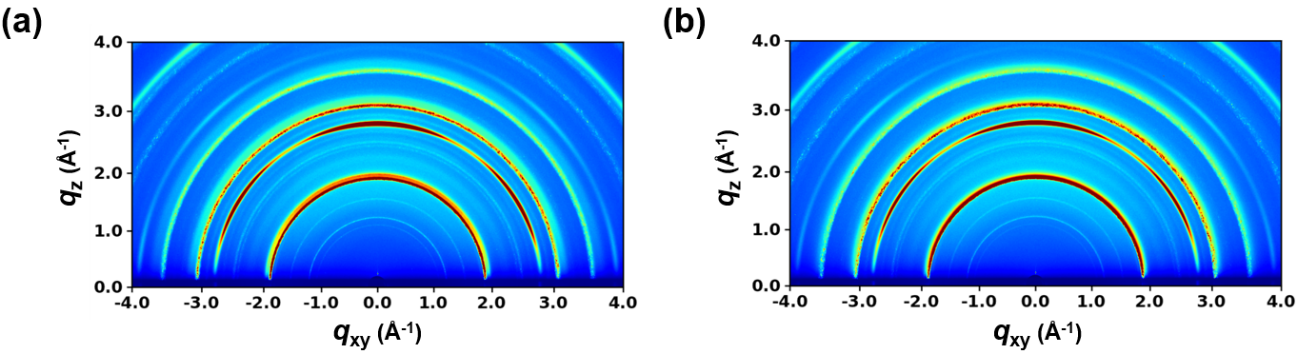


**Figure S2.** 2D GI-WAXS patterns of the (a) solCIGS and (b) K-solCIGS thin films.


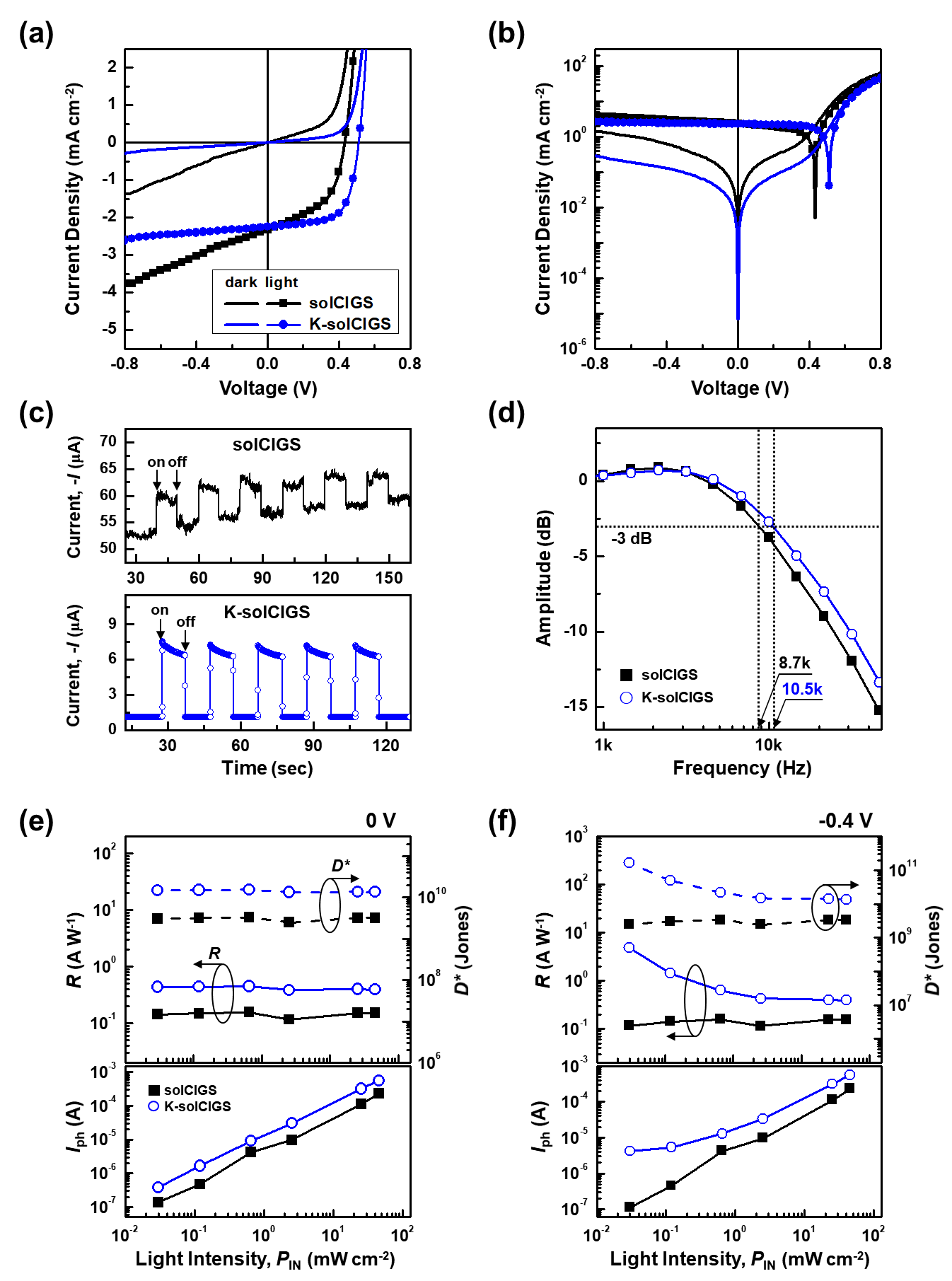


**Figure S3.** (a,b) Current density‒voltage (*J*‒*V*) characteristics of solCIGS and K-solCIGS photodiodes in the dark and under illumination at the NIR wavelength of 850 nm (*P*_IN_ = 45.5 mW cm^-2^). Note that the graphs in (a) and (b) show a linear plot and a semi-logarithmic plot of the *J*‒*V* characteristics, respectively. (c) Time-dependent photoresponse of solCIGS and K-solCIGS devices at -0.4 V (*λ* = 850 nm, *P*_IN_ = 0.03 mW cm^-2^). (d) -3 dB bandwidth comparison of solCIGS and K-solCIGS devices as a function of incident pulse laser frequency under NIR light of 850 nm. (e,f) Responsivity (*R*), detectivity (*D*^*^), and photocurrent (*I*_ph_) as functions of the light intensity (*P*_IN_) under NIR wavelength illumination of 850 nm at (e) 0 V and (f) -0.4 V.
